# Isotretinoin-induced myositis

**DOI:** 10.1093/omcr/omae017

**Published:** 2024-03-25

**Authors:** Oliver Hague, Vasiliki Thanopoulou, Pamela Mangat, Animesh Singh, Richard Stratton, Tamir Malley

**Affiliations:** Department of Rheumatology, Royal Free Hospital, London NW3 2QG, United Kingdom; Department of Rheumatology, Royal Free Hospital, London NW3 2QG, United Kingdom; Department of Rheumatology, Royal Free Hospital, London NW3 2QG, United Kingdom; Department of Rheumatology, Royal Free Hospital, London NW3 2QG, United Kingdom; Department of Rheumatology, Royal Free Hospital, London NW3 2QG, United Kingdom; Department of Rheumatology, Royal Free Hospital, London NW3 2QG, United Kingdom

## Abstract

Retinoid-induced myositis is a phenomenon recognised in multiple case reports. We report a case of isotretinoin-induced myositis in an 18-year-old male patient. This case adds to the published literature as it demonstrates (i) myositis may occur after extended periods of isotretinoin use, (ii) should be considered as a differential diagnosis even when presenting asymmetrically and (iii) can continue to progress clinically and biochemically initially following the suspension of isotretinoin before being effectively treated with corticosteroids.

## INTRODUCTION

Isotretinoin is a first-generation vitamin A derived retinoid medication used in follicular dermatological diseases and skin lesions associated with systemic disease, for example acne vulgaris and discoid lupus erythematous, respectively. Isotretinoin is thought to induce apoptosis in cell lineages which convert it to all-trans retinoic acid, including sebatocytes but also hepatocytes, intestinal epithelium and myocytes [1].

Isotretinoin is associated with a range of side effects (Table 1). Musculoskeletal side effects are the most common, reported to occur in 49.8% of patients in a recent report of 200 patients receiving isotretinoin over 6–12 months of treatment [2]. Clinical, biochemical and imaging characteristics are diverse. Published cases range from asymptomatic elevation in markers of muscle break down, such as creatine kinase (CK), to focal (including extraocular myositis) or generalised myositis, and even to severe necrotising myositis and rhabdomyolysis requiring intensive care, and may potentially be fatal [3].

**Table 1:** Reported Side-Effects of Isotretinoin

Body System	Reported Side Effects
Musculoskeletal	Back pain, Myalgia, Myositis, Arthralgia, Spondylarthritis, Enthesitis, Rhabdomyolysis
Dermatological	Sun sensitivity, Hair loss, Facial erythema, Xerosis, Dermatitis, Cheilitis
Neurological/Psychiatric	Altered vision, Headache, Insomnia, Low mood, Suicidal Ideation
Other	Abdominal pain, Epistaxis, Menorrhagia, Polydipsia

Side effects usually occur in the first month after initiation of therapy [4]. While studies show male sex [5] and possession of specific polymorphisms in cell-death cascade proteins are associated with elevated CK titres and symptomatic arthralgia [6], respectively, there is limited understanding of other determinants of susceptibility to these side-effects.

## CASE REPORT

An 18-year-old male patient with a background of ulcerative colitis and primary sclerosing cholangitis (treated with mesalamine and ursodeoxycholic acid, respectively) presented with acute onset, atraumatic, non-exertional right calf pain, followed closely by right buttock pain, 18 months after commencing isotretinoin for nodulocystic acne. Aside from a 3-month period when isotretinoin was temporarily withheld due to deranged liver function tests, there was full concordance with treatment. On initial assessment in the Emergency Department his vital signs were normal. Bloods results: haemoglobin *95 g/l* (135–170), white cell count 15.54 × 10^*^9/l (3.5–11), neutrophils 11.48 × 10^*^9/l (1.7–7.5), ALT 89 U/l (10–50), AST 128 U/l (0–50), CRP 55 mg/l (0–5), CK 3905 mg/l (39–308). Viral serology was negative for HIV, and hepatitis B and C. He was diagnosed with myositis and initially treated with analgesia, fluids and isotretinoin was discontinued. He was discharged with follow-up later that week.

**Figure 1:**
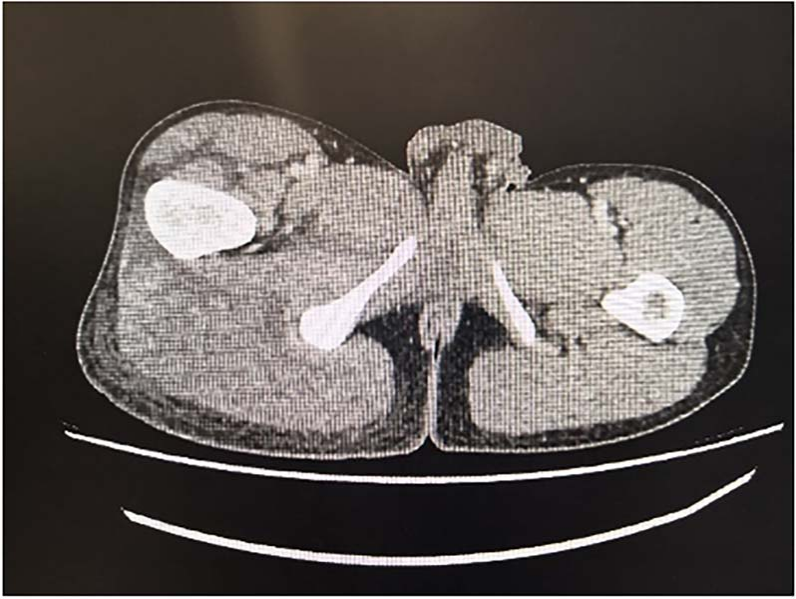
CT pelvis demonstrating asymmetric expansion of the right gluteus maximum muscle and associated oedema/stranding in keeping with muscle oedema. Fluid seen tracking into the posterior and lateral compartments of the thigh.

Over the next three days, he developed pain over the left shoulder and weakness in both lower limbs with an inability to weight-bear. He returned to hospital prior to the scheduled follow-up appointment. On examination there was reduced power in right hip flexion and extension (MRC grade 3/5), knee flexion and extension (MRC grade 4/5) and in left shoulder abduction, adduction, flexion and extension (MRC grade 4/5). There was tenderness over the right thigh, right buttock and left deltoid. There was no erythema, rashes or other stigmata of dermatomyositis. Repeat bloods: haemoglobin 103 *g/l* (135–170), white cell count 24.49 × 10^*^9/l (3.5–11), ALT 70 U/l (−50), ALP 156 U/l (30–130), CRP 228 mg/l (0–5), CK 4331 mg/l(39–308). Immunology and myositis antibody panels were negative. CT pelvis demonstrated asymmetric expansion and oedema of the right gluteus maximus muscle with associated tracking of fluid into the posterior and lateral compartments of the thigh (Fig. 1).

**Figure 2:**
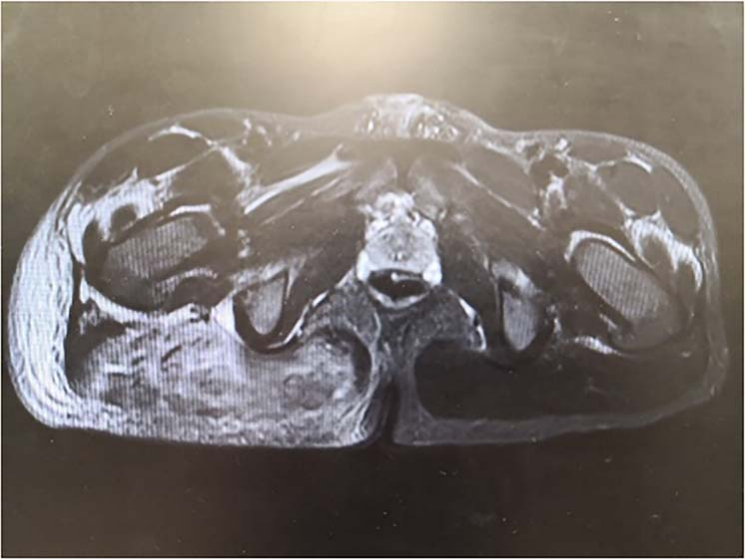
MRI pelvis demonstrating extensive inflammation of the right gluteus maximus with subcutaneous oedema.

Piperacillin-tazobactam and clindamycin were commenced to empirically cover for necrotising fasciitis and infective myositis. MRI demonstrated extensive inflammation of the right gluteus maximus with subcutaneous oedema (Fig. 2). Electromyography showed patchy myopathic motor units in the upper and lower limb muscles, fibrillations and positive sharp waves in some of these muscle groups indicative of muscle membrane instability. Nerve conduction studies were normal with no evidence of generalised large fibre polyneuropathy.

**Figure 3:**
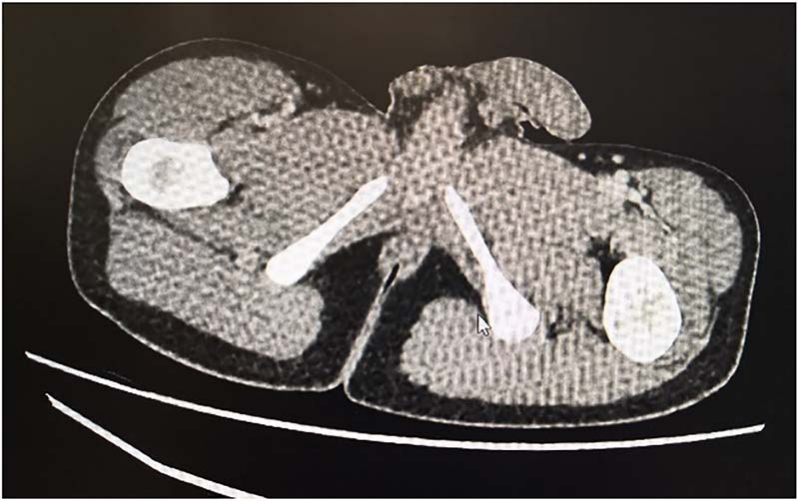
CT demonstrating resolution of the right gluteus maximus oedema.

Following these investigations, a diagnosis of myositis was confirmed. On the advice of the infectious diseases team, a 7-day course of antibiotics was completed, although an infectious trigger was not found, nor did antimicrobial therapy improve his clinical or biochemical features. With drug-induced myositis (secondary to isotretinoin) emerging as the most likely diagnosis, a course of Prednisolone was commenced to hasten his recovery. His pain improved significantly and there was a rapid reduction in CK to 259 mg/l (39–308). He was discharged with a weaning course of prednisolone. He weaned off steroids within 2 months with sustained normalisation of CK and CRP. CT abdomen and pelvis performed for a separate indication 5 months later incidentally revealed resolution of the right gluteus maximum oedema (Fig. 3).

## DISCUSSION

There are 9 published reports of myositis attributed to isotretinoin use (as summarised by Rivillas et al [3]). In these cases, onset of symptoms occurred between 10–90 days (mean of 42 days) of starting isotretinoin, thus 18 months of therapy represents a substantially longer duration of treatment prior to development of myositis. To our knowledge, this is the first reported case to present with markedly asymmetric symptoms. Myositis can be identified and localised in multiple ways. Clinically, by the presence of pain and/or weakness, as well as evidence of muscular oedema and changes in fat saturation on MRI, which can provide clues as to the aetiology of the myositis and guide sites for biopsy [7, 8].

Suspicion of isotretinoin-induced myositis should lead to discontinuation of isotretinoin. Despite doing so in this case, there was progression of pain and weakness, spreading of involved muscles and continued rise in CRP and CK. The clinical and biochemical progression despite cessation of isotretinoin emphasises the importance of multi-speciality input and investigation to exclude other serious manifestations, for example necrotising fasciitis. A diagnosis of isotretinoin-induced myositis was reached based on the absence of an alternative identifiable cause, alongside compatible imaging and electrophysiological findings.

Symptoms markedly improved on initiation of corticosteroid therapy, with complete and sustained resolution following completion of a weaning regime. There have been no previous reports of corticosteroid or disease-modifying-antirheumatic-drugs use in the treatment of isotretinoin-induced myositis [3]. In selected cases of drug-induced myopathies, such as following statin exposure, removal of causative medication has been noted to be insufficient, requiring the use of immunosuppression [9].
